# Comparative efficacy and safety of anticoagulant, combination, and antiplatelet therapies in patients with intracardiac thrombus: a real-world retrospective study

**DOI:** 10.3389/fmed.2026.1723890

**Published:** 2026-02-18

**Authors:** Chenxiao Jiang, Xinyu Wang, Baoyan Wang, Xin Chen, Biao Xu, Jie Ni, Dujuan Sha

**Affiliations:** 1 Department of Pharmacy, Nanjing Drum Tower Hospital, Affiliated Hospital of Medical School, Nanjing University, Nanjing, Jiangsu, China; 2 Department of General Medicine, Nanjing Drum Tower Hospital Clinical College of Nanjing University of Chinese Medicine, Nanjing, Jiangsu, China; 3 Department of Cardiology, Nanjing Drum Tower Hospital, Affiliated Hospital of Medical School, Nanjing University, Nanjing, Jiangsu, China; 4 Department of General Medicine, Nanjing Drum Tower Hospital, Affiliated Hospital of Medical School, Nanjing University, Nanjing, Jiangsu, China

**Keywords:** anticoagulant therapy, antiplatelet therapy, combination therapy, efficacy, intracardiac thrombus, safety

## Abstract

**Background:**

Intracardiac thrombus is associated with significant morbidity and mortality; however, optimal antithrombotic strategies remain uncertain.

**Objectives:**

To evaluate the efficacy and safety of different antithrombotic regimens in patients with intracardiac thrombus.

**Methods:**

In this retrospective study, patients with intracardiac thrombus were enrolled at Nanjing Drum Tower Hospital. Logistic regression assessed the association between antithrombotic therapies and thrombus dissolution, while Cox regression evaluated the association with all-cause mortality. Thromboembolic events and clinically related major bleeding (CRMB) events were compared across treatment groups using chi-square or Fisher’s exact tests. Kaplan–Meier curves depicted one-year cumulative incidence, with subgroup analyses presented using forest plots.

**Results:**

A total of 214 patients were included, of whom 26 received antiplatelet therapy, 118 anticoagulant therapy, and 70 combination therapy. Compared with antiplatelet therapy, anticoagulant therapy was associated with a significantly higher likelihood of thrombus dissolution in the Model 1 (aOR 3.56, 95% CI 1.16–11.20), although this association was attenyated in Model 2 (aOR 2.52, 95% CI: 0.72–9.01). Combination therapy showed a non-significant trend toward improved thrombus dissolution in both Models 1 (aOR 2.40, 95% CI 0.77–7.37) and Model 2 (aOR 2.12, 95% CI 0.66–6.66). For all-cause mortality, anticoagulant therapy was consistently associated with a significantly lower risk compared with antiplatelet therapy in both adjusted models (Model 1: aHR 0.20, 95% CI 0.05–0.79; Model 2: aHR 0.16, 95% CI 0.03–0.78). Compared with antiplatelet therapy, combination therapy was associated with reduced all-cause mortality in the Model 1 (aHR 0.16, 95% CI 0.03–0.90) but not Model 2 (aHR 0.34, 95% CI 0.06–1.75). Combination therapy showed a non-significant trend toward improved thrombus dissolution or all-cause mortality compared with anticoagulant therapy.

**Conclusion:**

In patients with intracardiac thrombus, anticoagulant monotherapy was associated with a significantly lower one-year all-cause mortality compared with antiplatelet therapy, whereas its effect on thrombus dissolution was attenuated after full adjustment, potentially reflecting thrombus-related heterogeneity. Combination therapy did not confer additional efficacy or safety benefits over antiplatelet or anticoagulant monotherapy.

## 1 Introduction

Intracardiac thrombus, a common cardiac mass lesion, is characterized by clot formation in one or more heart chambers, particularly the left ventricle (LV), left atrial appendage (LAA), and atrial. The development of intracardiac thrombus is frequently associated with conditions such as myocardial infarction (MI), heart failure (HF), atrial fibrillation (AF), and dilated cardiomyopathy (DCM). Given its strong correlation with these cardiovascular diseases and the risk of thromboembolic events, intracardiac thrombus has become a significant focus of clinical attention. These underlying diseases contribute to a triad of pathophysiological changes, including slowed venous blood flow, hypercoagulability, and endothelial injury, that collectively promote blood stasis and thrombus formation (1). Among intracardiac thrombus, left ventricular thrombus (LVT) has been reported in 7–46% of patients after MI, depending on population characteristics and diagnostic criteria (2). In AF, thrombus formation predominantly occurs in the LAA, where up to 91% of atrial thrombus originates (3).

Intracardiac thrombus poses significant clinical challenges due to its strong association with systemic embolism, including MI, ischemic stroke, and venous thromboembolism (VTE) (4). Beyond its embolic potential, it is linked to poor prognosis and increased mortality (5). Current treatment strategies vary widely and are heavily influenced by the thrombus type and the underlying cardiac condition. For LVT, both LVT-specific guidelines and ST-segment elevation myocardial infarction (STEMI) guidelines recommend oral anticoagulants (OACs) as the primary therapy (6, 7). However, real-world registries show that a small but notable proportion of patients with LVT continue to receive antiplatelet therapy alone (8, 9), and some receive combination therapy with both anticoagulants and antiplatelet agents (10–12). In contrast, for atrial or LAA thrombus, there is a lack of clear guideline recommendations for antithrombotic therapy, though some studies support the use of OACs in these scenarios (13, 14). Despite the clinical relevance of antithrombotic therapy in patients with intracardiac thrombus, most existing studies have focused on a single thrombus subtype or have compared only two antithrombotic strategies. As a result, real-world evidence directly comparing antiplatelet therapy, anticoagulation, and combination therapy within the same cohort and across different intracardiac thrombus types remains scarce.

Accordingly, this study was designed to systematically compare the efficacy and safety of three commonly used antithrombotic strategies in a real-world cohort of patients with different types of intracardiac thrombus. Specifically, we sought to evaluate whether anticoagulation-based strategies (including anticoagulation alone or in combination with antiplatelet therapy) were associated with improved thrombus dissolution and lower all-cause mortality compared with antiplatelet therapy alone, without a corresponding increase in thromboembolic or clinically relevant bleeding events. Given the limited and inconsistent evidence regarding additional benefit from combination therapy, no *a priori* assumption was made regarding its superiority over anticoagulation alone.

## 2 Methods

### 2.1 Study design and population

This retrospective cohort study involved patients with a first-time diagnosis of intracardiac thrombus (LVT, atrial, or LAA thrombus) at Nanjing Drum Tower Hospital between January 2018 and December 2024. Patients were excluded if they met any of the following criteria: (1) missing baseline data; (2) missing echocardiographic data; (3) lack of antithrombotic drugs during hospitalization; (4) undergoing thrombectomy during hospitalization; or (5) loss to follow-up.

Patients were divided into three groups based on discharge medications: the antiplatelet group, the anticoagulant group, and the combination group. In the antiplatelet group, patients received aspirin, clopidogrel, or ticagrelor as single antiplatelet therapy (SAPT) or dual antiplatelet therapy (DAPT). The anticoagulant group comprised patients prescribed warfarin, rivaroxaban, dabigatran, or edoxaban. The combination group included those discharged on one or two antiplatelet drugs plus an oral anticoagulant. Pairwise comparisons—antiplatelet versus anticoagulant, antiplatelet versus combination, and anticoagulant versus combination—were then performed to evaluate differences in primary efficacy and safety outcomes.

### 2.2 Baseline data collection

Baseline characteristics of patients were collected, including demographic details, medical and medication history, echocardiographic indicators, types of thrombus and initial laboratory parameters.

### 2.3 Outcome assessment

The follow-up period for this study was 1 year. The primary endpoints were thrombus dissolution and all-cause mortality during follow-up. Intracardiac thrombus was confirmed by transthoracic or transesophageal echocardiography (TTE/TEE) during the index hospitalization. Thrombus resolution was assessed by follow-up TTE or TEE performed within 1 year after treatment initiation, according to routine clinical practice. Given the retrospective design of the study, thrombus resolution was defined that the thrombus was described as reduced in size or completely absent compared with baseline imaging, based on the descriptive findings documented in the finalized echocardiography reports. All echocardiographic examinations were reviewed as part of routine clinical reporting by echocardiographers and clinicians. Image interpretation was not blinded to clinical information. Secondary outcomes included CRMB events and thromboembolic events during follow-up. CRMB events were defined according to the International Society on Thrombosis and Hemostasis criteria (ISTH) (15). CRMB is defined as fatal bleeding, and/or symptomatic bleeding in a critical area or organ, such as intracranial, intraspinal, intraocular, retroperitoneal, intra-articular or pericardial, or intramuscular with compartment syndrome, and/or bleeding causing a fall in hemoglobin level of 20gL^−1^ (1.24 mmolL^−1^) or more, or leading to transfusion of two or more units of whole blood or red cells. Thromboembolic events were defined as systemic embolism, stroke, MI, and recurrence of intracardiac thrombus.

### 2.4 Statistics analysis

All analyses were conducted using R software (version 4.3.3). Continuous variables with a normal distribution were presented as mean ± standard deviation (SD), while non-normal variables were presented as median and interquartile range (IQR). Student’s *t*-test was used for comparison of continuous variables, and categorical variables were compared using the Chi-Square test or Fisher’s exact test.

Logistic regression models were applied to estimate odds ratios (ORs) and 95% confidence intervals (CIs) for the association between antithrombotic strategies and thrombus resolution. Time-to-event analyses for all-cause mortality were performed using Cox proportional hazards models, yielding hazard ratios (HRs) with 95% CIs. Covariates entered into multivariable models were selected based on clinical relevance to the outcome or a *p* value < 0.10 in univariable analyses. Multicollinearity among covariates was assessed using variance inflation factors (VIFs), with all VIFs≤5 (16). Cumulative incidence curves for mortality, thromboembolic events, and clinically relevant non-major bleeding (CRMB) were generated via the Kaplan–Meier method and compared between groups using the log-rank test. Subgroup analyses, categorized by age (< 65 years vs. ≥ 65 years), coronary heart disease (CHD), and heart failure (HF), were conducted to explore these relationships further. The results were visually represented using forest plots. Statistical significance was *p* ≤ 0.05, with *p*-values for interaction exceeding 0.1 indicating a lack of significant interaction.

This study followed the Declaration of Helsinki and received approval from the Human Ethical Committee and Medical Research Council of Nanjing Drum Tower Hospital (Ethic Number: 2024-493-02).

## 3 Results

### 3.1 Patient population

**Figure 1:**
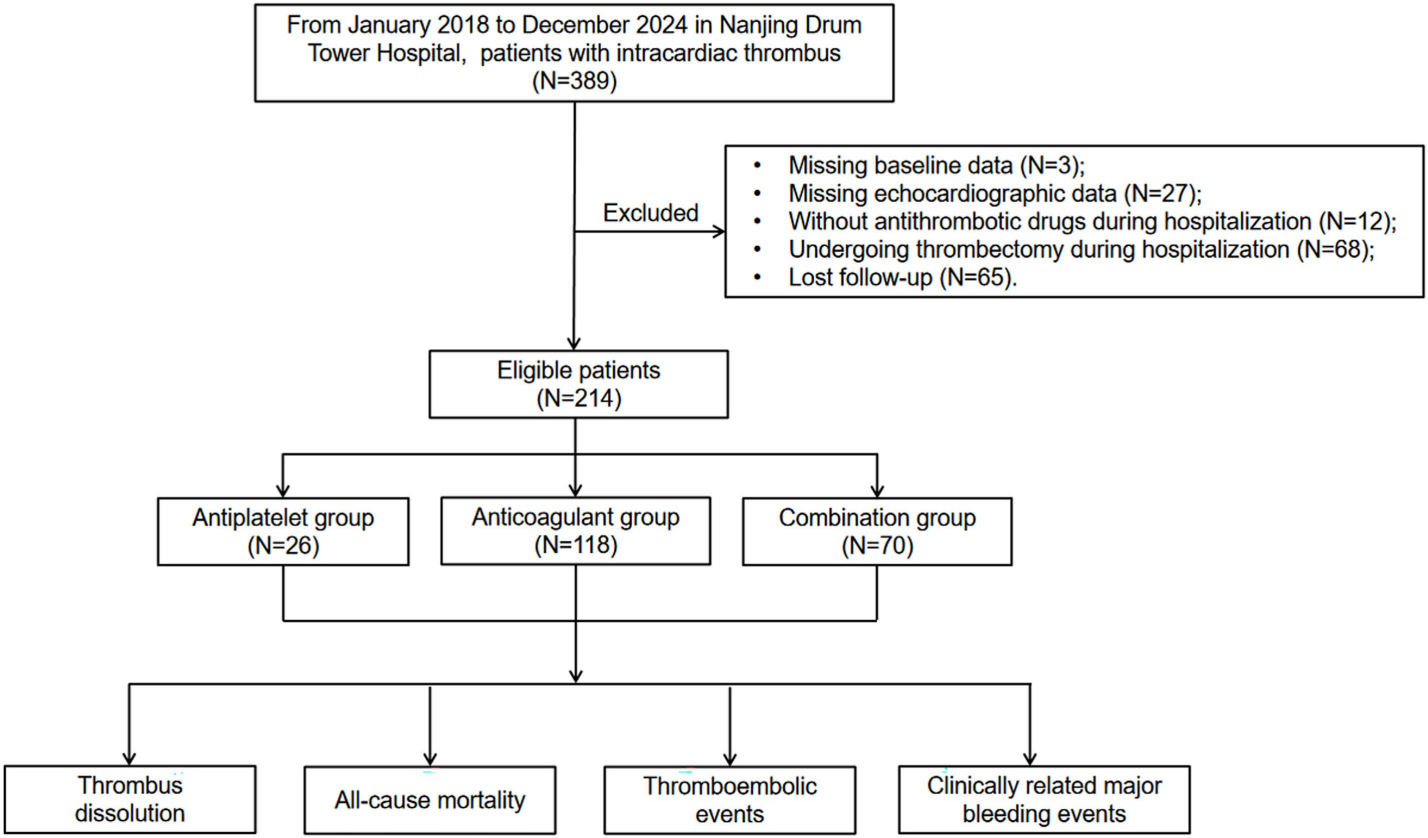
Flow diagram of study patients.

**Figure 2:**
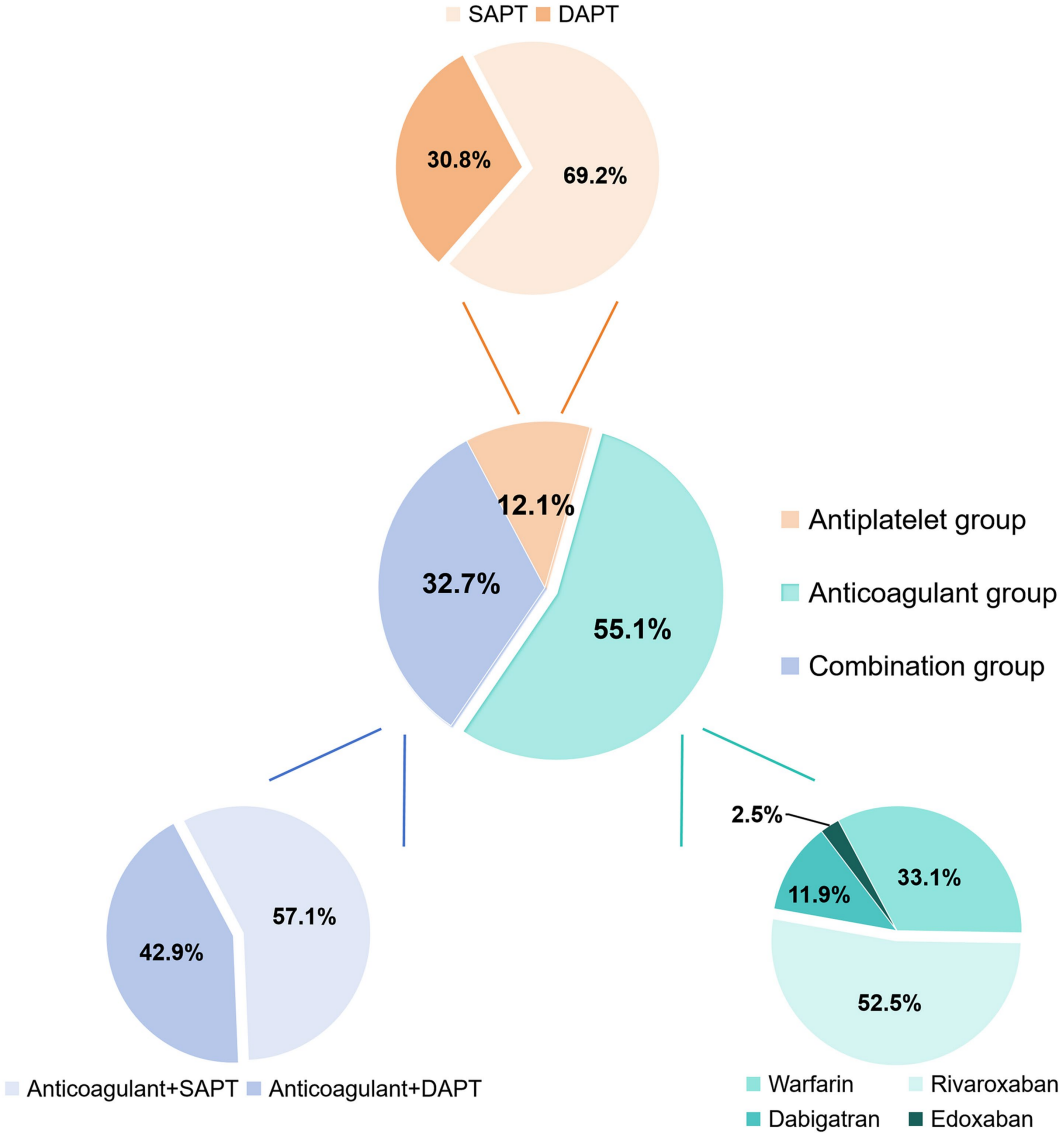
Grouping of treatment protocols for antithrombotic therapies. SAPT, single antiplatelet therapy; DAPT, dual antiplatelet therapy.

A total of 389 patients with intracardiac thrombus at Nanjing Drum Tower Hospital from January 2018 to December 2024 were initially included in the study (Figure 1). Of these, 175 patients were excluded due to missing baseline data (*n* = 3), missing echocardiographic data (*n* = 27), absence of antithrombotic drugs during hospitalization (*n* = 12), undergoing thrombectomy during hospitalization (*n* = 68) and lost to follow-up (*n* = 65). Finally, 214 patients were enrolled in the final analysis, with 12.1% (26/214) patients in the antiplatelet group, 55.1% (118/214) in the anticoagulant group, and 32.7% (70/214) in the combination group (Figure 2).

Antithrombotic regimens were further categorized according to specific treatment strategies and dosing patterns. In the antiplatelet group, 18 patients (69.2%) received SAPT and 8 (30.8%) received DAPT. In the anticoagulant group, warfarin was prescribed to 39 (33.1%) patients, rivaroxaban to 62 (52.5%), dabigatran to 14 (11.9%), edoxaban to 3 (2.5%). In the combination group, 40 (57.1%) patients received anticoagulants with SAPT, while 30 (42.9%) received anticoagulants with DAPT. Detailed medication regimens are provided in Supplementary Tables 1–3.

### 3.2 Baseline characteristics

Table 1 presents the baseline characteristics of the patients with intracardiac thrombus. Patients receiving anticoagulant therapy were more frequently female and had a higher mean body mass index (BMI). In contrast, they exhibited a lower prevalence of coronary heart disease (CHD), renal insufficiency, and malignancy. Echocardiographic parameters in the anticoagulant group also exhibited greater interventricular septum thickness at end-diastole (IVSTd), left atrial diameter (LAD), left ventricular ejection fraction (LVEF). In addition, these patients had higher serum albumin (ALB) levels but lower platelet counts (PLT), C-reactive protein (CRP), and d-dimer (DD) levels. Compared with the antiplatelet group, the combination group exhibited a higher mean BMI, a lower proportion of patients with hyperuricemia and atrial fibrillation, and reduced of DD levels. No other baseline variables differed significantly between these two groups.

**Table 1:** Baseline characteristics of patients with intracardiac thrombus.

Variables	All	Antiplatelet group	Anticoagulant group	Combination group	*p*_1_	*p_2_*	*p_3_*
	*N* = 214	*N* = 26	*N* = 118	*N* = 70			
Demographic							
Female, *n* (%)	58 (27.1)	2 (7.7)	46 (39.0)	10 (14.3)	0.005	0.503	0.001
Age, y	64.5 (50.0, 71.0)	69.5 (52.0, 73.8)	64.0 (51.0, 70.0)	59.0 (47.3, 70.0)	0.191	0.129	0.383
BMI, kg/m^2^	23.9 (21.8, 26.3)	22.3 (20.8, 24.5)	24.1 (21.9, 26.7)	24.4 (22.2, 26.1)	0.032	0.029	0.828
History of smoking, *n* (%)	81 (37.9)	11 (42.3)	32 (27.1)	38 (54.3)	0.195	0.416	<0.001
History of drinking, *n* (%)	38 (17.8)	4 (15.4)	24 (20.3)	10 (14.3)	0.761	1.000	0.397
Medical history, *n* (%)							
Cardiac surgery	63 (29.4)	8 (30.8)	31 (26.3)	24 (34.3)	0.823	0.935	0.316
Hypertension	121 (56.5)	15 (57.7)	60 (50.8)	46 (65.7)	0.678	0.626	0.067
Hyperlipidemia	12 (5.6)	1 (3.8)	4 (3.4)	7 (10.0)	1.000	0.441	0.104
Hyperuricemia	14 (6.5)	4 (15.4)	8 (6.8)	2 (2.9)	0.230	0.044	0.326
Diabetes	61 (28.5)	9 (34.6)	27 (22.9)	25 (35.7)	0.317	1.000	0.083
Atrial fibrillation	33 (15.4)	4 (15.4)	27 (22.9)	2 (2.9)	0.563	0.044	0.001
Valve disease	3 (1.4)	0 (0.0)	3 (2.5)	0 (0.0)	1.000	1.000	0.295
CHD	54 (25.2)	11 (42.3)	18 (15.3)	25 (35.7)	0.004	0.722	0.002
Heart failure	39 (18.2)	6 (23.1)	22 (18.6)	11 (15.7)	0.808	0.387	0.755
Stroke	36 (16.8)	4 (15.4)	23 (19.5)	9 (12.9)	0.785	0.745	0.332
Hepatic insufficiency	11 (5.1)	3 (11.5)	4 (3.4)	4 (5.7)	0.111	0.384	0.473
Renal insufficiency	14 (6.5)	5 (19.2)	5 (4.2)	4 (5.7)	0.018	0.058	0.729
Bleeding	9 (4.2)	2 (7.7)	6 (5.1)	1 (1.4)	0.636	0.177	0.261
Malignancy	11 (5.1)	3 (11.5)	2 (1.7)	6 (8.6)	0.041	0.700	0.054
Medication history, *n* (%)							
Antithrombotic drug	31 (14.5)	5 (19.2)	20 (16.9)	6 (8.6)	0.778	0.162	0.164
Antihypertensive drug	93 (43.5)	12 (46.2)	46 (39.0)	35 (50.0)	0.650	0.916	0.186
Lipid-lowering drug	13 (6.1)	2 (7.7)	7 (5.9)	4 (5.7)	0.665	0.661	1.000
Hypoglycemic drug	44 (20.6)	4 (15.4)	18 (15.3)	22 (31.4)	1.000	0.189	0.015
Echocardiographic indicators							
IVSTd, cm	0.8 (0.7, 1.0)	0.8 (0.7, 0.8)	0.9 (0.8, 1.0)	0.8 (0.7, 1.0)	0.009	0.156	0.106
LVDd, cm	5.6 (5.0, 6.2)	5.7 (5.6, 6.5)	5.6 (4.9, 6.3)	5.4 (5.1, 6.0)	0.171	0.069	0.849
LVPWTd, cm	0.9 (0.8, 1.0)	0.9 (0.8, 0.9)	0.9 (0.8, 1.0)	0.9 (0.8, 1.0)	0.488	0.544	0.930
AoD, cm	3.2 (2.9, 3.4)	3.2 (3.1, 3.3)	3.2 (2.9, 3.5)	3.1 (2.9, 3.3)	0.564	0.413	0.076
LAD, cm	4.4 (4.0, 4.9)	4.2 (4.0, 4.4)	4.7 (4.3, 5.4)	4.2 (3.8, 4.5)	<0.001	0.527	<0.001
LVEF, %	41.0 (34.0, 53.0)	38.5 (31.5, 41.0)	45.5 (31.3, 57.0)	40.0 (36.0, 46.8)	0.020	0.087	0.046
PASP, mmHg	35.0 (30.0, 40.0)	35.5 (26.3, 40.0)	36.0 (31.3, 45.0)	30.0 (25.0, 36.8)	0.201	0.109	<0.001
Thrombus types, *n* (%)							
Atrial thrombus	39 (18.2)	0 (0.0)	34 (28.8)	5 (7.1)	<0.001	0.225	<0.001
Left ventricular thrombus	126 (58.9)	24 (92.3)	39 (33.1)	63 (90.0)			
Left atrial appendage thrombus	49 (22.9)	2 (7.7)	45 (38.1)	2 (2.9)			
Laboratory examinations							
PLT, 10^^9^/L	182.0 (143.0, 225.8)	206.5 (167.5, 251.3)	171.5 (132.0, 210.0)	195.5 (166.0, 240.8)	0.032	0.671	0.006
Hb, g/L	136.5 (126.3, 150.0)	132.5 (121.0, 148.8)	136.0 (126.3, 149.0)	139.0 (130.0, 152.8)	0.506	0.165	0.254
Cr, μmol/L	77.0 (64.0, 93.8)	81.5 (71.3, 94.8)	75.5 (60.0, 88.8)	76.5 (64.3, 101.8)	0.122	0.553	0.233
GFR, ml/min	89.6 (71.4, 108.6)	87.3 (71.9, 100.2)	90.2 (73.8, 106.2)	95.5 (67.5, 116.5)	0.482	0.606	0.845
CRP, mg/L	6.3 (3.3, 23.4)	20.0 (6.6, 29.0)	4.7 (2.9, 12.8)	12.4 (5.1, 54.0)	0.003	0.947	<0.001
TG, mmol/L	1.1 (0.9, 1.5)	1.2 (1.0, 1.4)	1.1 (0.8, 1.4)	1.2 (0.9, 1.8)	0.191	0.726	0.032
TC, mmol/L	3.8 (3.2, 4.6)	3.9 (3.2, 4.5)	3.6 (3.1, 4.5)	4.1 (3.4, 5.3)	0.439	0.438	0.021
ALB, g/L	39.1 (36.2, 41.1)	36.3 (33.8, 39.8)	39.3 (36.8, 41.2)	39.2 (35.5, 41.1)	0.007	0.071	0.535
HDLC, mmol/L	1.0 (0.8, 1.2)	0.9 (0.8, 1.0)	1.0 (0.8, 1.2)	0.92 (0.8, 1.1)	0.244	0.689	0.309
LDLC, mmol/L	2.2 (1.7, 2.8)	2.3 (1.6, 2.7)	2.1 (1.7, 2.7)	2.4 (1.8, 3.3)	0.461	0.382	0.021
PT, s	12.1 (11.5, 13.3)	12.1 (11.5, 13.0)	12.3 (11.6, 13.8)	11.7 (11.2, 12.7)	0.134	0.278	0.001
INR	1.1 (1.0, 1.2)	1.1 (1.0, 1.2)	1.1 (1.0, 1.2)	1.0 (1.0, 1.1)	0.115	0.246	<0.001
APTT, s	27.9 (26.0, 30.6)	27.1 (25.6, 29.3)	28.1 (26.3, 30.7)	28.2 (26.0, 30.8)	0.197	0.253	0.949
TT, s	17.9 (17.1, 18.9)	17.7 (16.8, 18.6)	18.1 (17.3, 19.1)	17.8 (16.9, 18.6)	0.157	0.879	0.095
FIB, g/L	3.1 (2.5, 3.9)	3.2 (2.7, 4.2)	2.8 (2.4, 3.4)	3.4 (2.7, 5.0)	0.081	0.380	<0.001
DD, mg/L	1.05 (0.32, 2.21)	2.21 (0.94, 3.92)	0.8 (0.3, 1.9)	0.9 (0.3, 2.2)	0.001	0.015	0.357

BMI, body mass index; CHD, coronary heart disease; IVSTd, interventricular septum thickness in diastole; LVDd, left ventricular diastolic diameter; LVPWTd, left ventricle posterior wall thickness in diastole; AoD, ascending aorta diameter; LAD, left atrial diameter; LVEF, left ventricular ejection fraction; PASP, pulmonary artery systolic pressure; PLT, platelet; Hb, hemoglobin; Cr, creatinine; GFR, glomerular filtration rate; CRP, C-reactive protein; TG, triglyceride; TC, total cholesterol; ALB, albumin; HDLC, high-density lipoprotein cholesterol; LDLC, low-density lipoprotein cholesterol; PT, prothrombin time; INR, international standardized ratio; APTT, activated partial thromboplastin time; TT, thrombin time; FIB, fibrinogen; DD, d-dimer. *p*_1_ is the *p* value of comparison between the anticoagulant group and the antiplatelet group. *p*_2_ is the *p* value of comparison between the combination group and the antiplatelet group. *p*_3_ is the *p* value of comparison between the combination group and the anticoagulant group.

Compared to the anticoagulant group, the combination group included fewer women and fewer individuals with atrial fibrillation, while exhibiting a higher prevalence of smoking, coronary heart disease, and use of hypoglycemic agents. This group also demonstrated a distinct laboratory and echocardiographic profile, with higher levels of PLT, CRP, triglyceride (TG), total cholesterol (TC), low-density lipoprotein cholesterol (LDLC) and fibrinogen (FIB), accompanied by lower LAD, LVEF, pulmonary artery systolic pressure (PASP), prothrombin time (PT) and international standardized ratio (INR).

In terms of thrombus types, LVT was predominant in both the antiplatelet (92.3%) and combination groups (90.0%). In contrast, the anticoagulant group showed a more heterogeneous pattern, comprising atrial thrombus (28.8%), LVT (33.1%), and LAA thrombus (38.1%).

### 3.3 Thrombus dissolution

**Figure 3:**
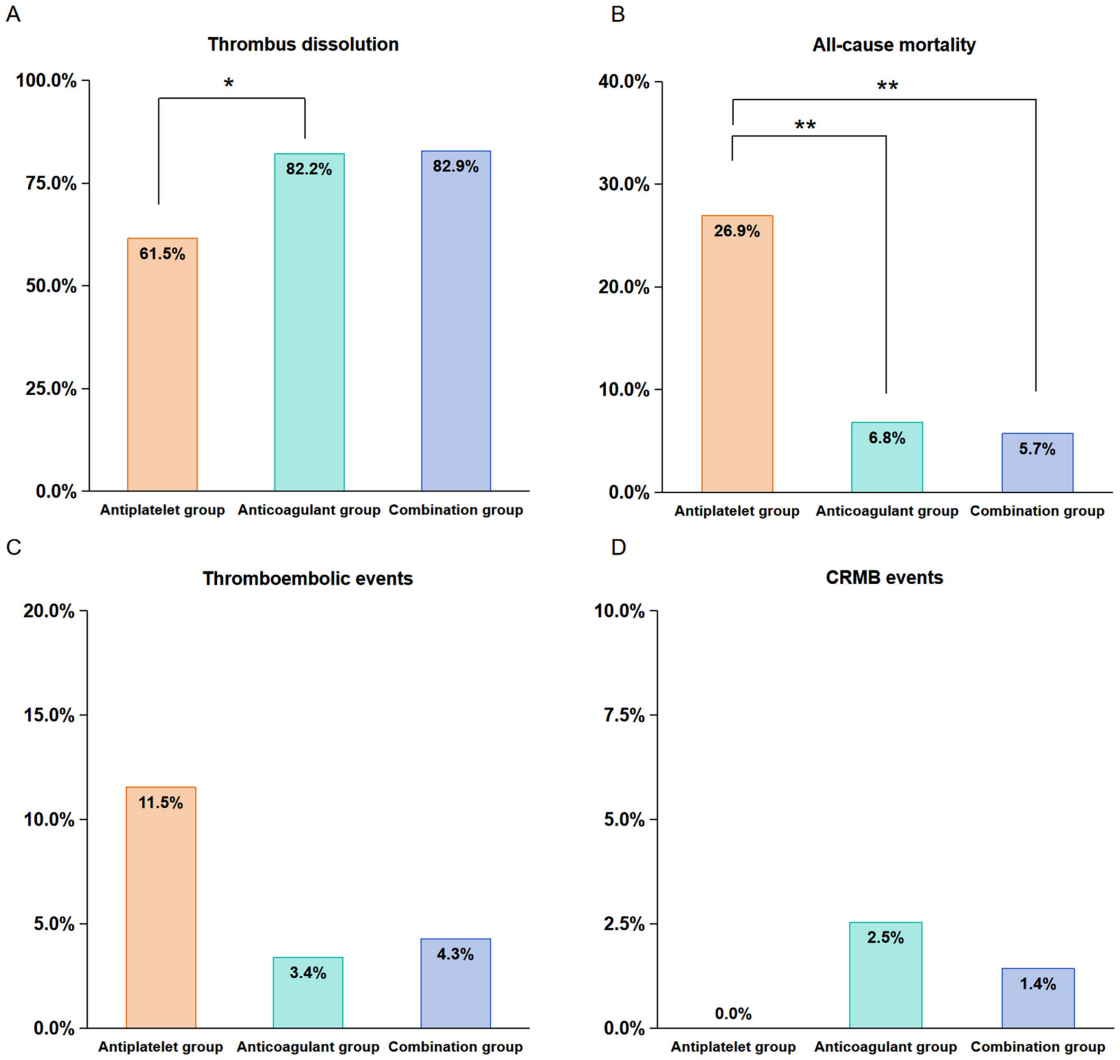
Incidences of thrombus dissolution **(A)**, all-cause mortality **(B)**, thromboembolic events **(C)**, and CRMB events **(D)** among the three treatment groups. CRMB, clinically related major bleeding.

At the 1-year follow-up, the thrombus dissolution rates were higher in the anticoagulant (82.2%) and the combination groups (82.9%) than in the antiplatelet group (61.5%) (Figure 3A) (anticoagulant vs. antiplatelet: *p* = 0.040; combination vs. antiplatelet: *p* = 0.053; combination vs. anticoagulant: *p* = 1.000). Echocardiographic examples at baseline and follow up are shown in Supplementary Figure 1. The thrombus resolution rate of patients with LAA thrombus (95.6%) in the anticoagulation group was higher than that of patients with atrial thrombus (67.6%) and LVT (79.5%) (LAA vs. atrial: *p* < 0.001; LAA vs. LVT: *p* = 0.039; LVT vs. atrial: *p* = 0.377; Supplementary Table 4). When stratified by thrombus subtype, no statistically significant differences in thrombus dissolution rates were detected among treatment groups within each thrombus category (Supplementary Table 5). These findings should be interpreted cautiously given the limited sample sizes in certain subgroups.

In univariate logistic regression, anticoagulant therapy (OR 2.89; 95% CI 1.15–7.25; *p* = 0.024) and combination therapy (OR 3.02; 95% CI 1.11–8.26; *p* = 0.031) were each associated with higher odds of thrombus resolution versus antiplatelet therapy, whereas there was no significant difference between combination and anticoagulant regimens (OR 1.05; 95% CI 0.48–2.28; *p* = 0.909) (Table 2).

**Table 2:** Logistic regression analysis of thrombus dissolution.

Group	Univariate analysis		Multivariate analysis			
			Model 1		Model 2	
	OR (95% CI)	*p* value	aOR (95% CI)	*p* value	aOR (95% CI)	*p* value
Part A: Antiplatelet group as the reference						
Antiplatelet group	Ref	–	Ref	–	Ref	–
Anticoagulant group	2.89 (1.15–7.25)	0.024	3.56 (1.16–11.20)	0.027	2.52 (0.72–9.01)	0.146
Combination group	3.02 (1.11–8.26)	0.031	2.40 (0.77–7.37)	0.127	2.12 (0.66–6.66)	0.199
Part B: Anticoagulant group as the reference						
Anticoagulant group	Ref	–	Ref	–	Ref	–
Combination group	1.05 (0.48–2.28)	0.909	0.87 (0.36–2.15)	0.756	1.52 (0.58–4.07)	0.393

Model 1 was adjusted for a history of cardiac surgery, atrial fibrillation, heart failure, age, sex, and BMI.

Model 2 was adjusted for thrombus types in addition to the variables in Model 1.

OR, odds ratio; aOR, adjusted odds ratio; 95% CI, 95% confidence interval.

In multivariate models logistic regression adjusted for a history of cardiac surgery, AF, HF, age, sex and BMI (Model 1), anticoagulant therapy was associated with a significantly higher likelihood of thrombus dissolution compared with antiplatelet therapy (Model 1: aOR 3.56, 95% CI 1.16–11.20, *p* = 0.027). After further adjustment for thrombus type (Model 2), this association remained directionally favorable but was no longer statistically significant (aOR 2.52, 95% CI 0.72–9.01; *p* = 0.146). The combination group showed a similar direction toward improved thrombus dissolution compared with antiplatelet therapy; however, the associations did not reach statistical significance in either Model 1 (aOR 2.40, 95% CI 0.77–7.37, *p* = 0.127) and Model 2 (aOR 2.12, 95% CI 0.66–6.66, *p* = 0.199). In direct comparisons, no significant difference in thrombus dissolution was observed between combination therapy and anticoagulant monotherapy in either Model 1 (aOR 0.87, 95% CI 0.36–2.15; *p* = 0.756) or Model 2 (aOR 1.52, 95% CI 0.58–4.07; *p* = 0.393).

### 3.4 All-cause mortality

The proportion of all-cause mortality at follow-up was significantly lower in the anticoagulant group (6.8%) and the combination group (5.7%) compared to the antiplatelet group (26.9%) (Figure 3B) (anticoagulant vs. antiplatelet: *p* = 0.007; combination vs. antiplatelet: *p* = 0.008; combination vs. anticoagulant: *p* = 1.000).

Univariate Cox regression analysis demonstrated a significant association between treatment groups and all-cause mortality (Table 3). Specifically, the anticoagulant group had a reduced risk of mortality compared to the antiplatelet group (HR 0.22, 95% CI 0.08–0.60, *p* = 0.003), and the combination group also showed a reduced risk (HR 0.18, 95% CI 0.05–0.63, *p* = 0.007). No statistically significant difference was observed between the combination and the anticoagulant groups (HR 0.82, 95% CI 0.25–2.73, *p* = 0.748). In multivariate Cox regression analysis adjusting for CHD, HF, stroke, malignancy, age, sex, BMI, a history of smoking, renal insufficiency, and DD (Model 1), anticoagulant therapy was associated with a significantly lower risk of all-cause mortality compared with antiplatelet therapy (aHR 0.20, 95% CI 0.05–0.79, *p* = 0.022). A similar reduction in mortality risk was observed in the combination therapy group in Model 1 (aHR 0.16, 95% CI 0.03–0.90, *p* = 0.038). After further adjustment for thrombus type (Model 2), the association between anticoagulant therapy and reduced all-cause mortality remained statistically significant (aHR 0.16, 95% CI 0.03–0.78, *p* = 0.023). In contrast, the mortality benefit observed with combination therapy in Model 1 (aHR 0.16, 95% CI 0.03–0.90, *p* = 0.038) was attenuated and no longer statistically significant in the fully adjusted Model 2 (aHR 0.34, 95% CI 0.06–1.75, *p* = 0.196). Direct comparisons between anticoagulant monotherapy and combination therapy showed no significant differences in all-cause mortality in either model (Model 1: aHR 0.40, 95% CI 0.08–2.13, *p* = 0.285; Model 2: aHR 0.40, 95% CI 0.08–2.02, *p* = 0.267).

**Table 3:** Cox regression analysis of all-cause mortality.

Group	Univariate analysis		Multivariate analysis			
			Model 1		Model 2	
	HR (95% CI)	*p* value	aHR (95% CI)	*p* value	aHR (95% CI)	*p* value
Part A: Antiplatelet group as the reference						
Antiplatelet group	Ref	–	Ref	–	Ref	–
Anticoagulant group	0.22 (0.08–0.60)	0.003	0.20 (0.05–0.79)	0.022	0.16 (0.03–0.78)	0.023
Combination group	0.18 (0.05–0.63)	0.007	0.16 (0.03–0.90)	0.038	0.34 (0.06–1.75)	0.196
Part B: Anticoagulant group as the reference						
Anticoagulant group	Ref	–	Ref	–	Ref	–
Combination group	0.82 (0.25–2.73)	0.748	0.40 (0.08–2.13)	0.285	0.40 (0.08–2.02)	0.267

Model 1 was adjusted for a history of coronary heart disease, heart failure, stroke, malignancy, age, sex, BMI, a history of smoking, renal insufficiency, and d-dimer.

Model 2 was adjusted for thrombus types in addition to the variables in Model 1.

HR, hazard ratio; aHR, adjusted hazard ratio; 95% CI, 95% confidence interval.

**Figure 4:**
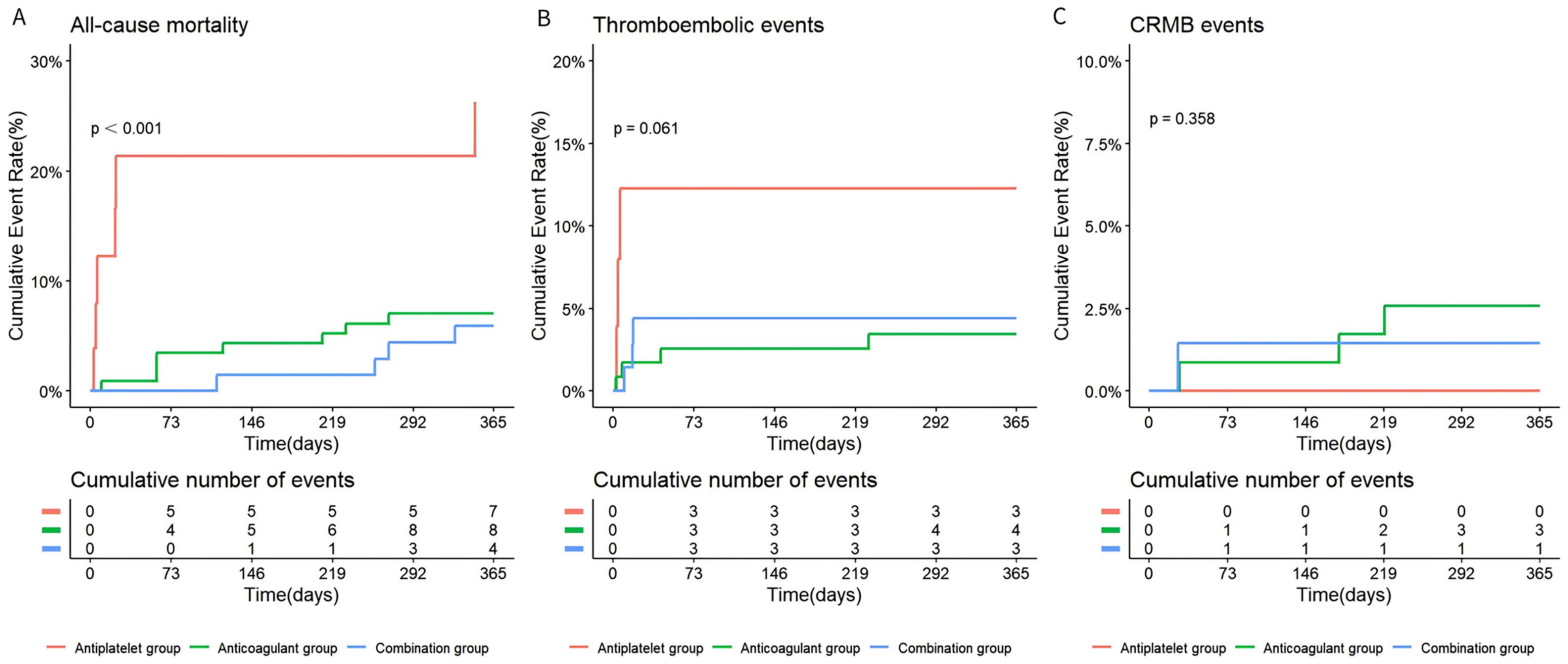
The Kaplan–Meier curves illustrating all-cause mortality **(A)**, thromboembolic events **(B)**, and CRMB events **(C)** among the three treatment groups during follow-up. Numbers at risk are shown below the survival curves at 0, 73, 146, 219, 292, and 365 days. CRMB, clinically related major bleeding.

Kaplan–Meier survival analysis revealed that the cumulative incidence of all-cause mortality was significantly higher in the antiplatelet group than in the anticoagulant and the combination groups (*p* < 0.001) (Figure 4A).

When stratified by treatment group, no statistically significant differences in all-cause mortality were detected among thrombus subtypes (Supplementary Table 6). When stratified by thrombus subtype, mortality rates varied across treatment groups (Supplementary Table 7). In patients with LV thrombus, lower mortality was observed in the combination group compared with the antiplatelet group (*p* = 0.024); however, this finding should be interpreted with caution given the small subgroup sizes and multiple comparisons. To more rigorously evaluate this association, we further conducted the univariate regression analysis on the LVT subgroup (Supplementary Table 8), and the results also supported that the combination treatment was a favorable factor for the reduction in all-cause mortality (HR 0.23, 95% CI 0.06–0.80, *p* = 0.021). Due to the small sample size of atrial thrombus (antiplatelet group: *n* = 0; anticoagulant group: *n* = 34; combination group: *n* = 5) and left atrial appendage thrombus (antiplatelet group: *n* = 2; anticoagulant group: *n* = 45; combination group: *n* = 2), it is not feasible to conduct regression analyses between these two subgroups.

### 3.5 Thromboembolic events

The incidence of thromboembolic events at follow-ups occurred in 11.5% (3/26) of the antiplatelet group, 3.4% (4/118) of the anticoagulant group, and 4.3% (3/70) of the combination group (anticoagulant vs. antiplatelet: *p* = 0.111; combination vs. antiplatelet: *p* = 0.339; combination vs. anticoagulant: *p* = 0.713) (Figure 3C). Kaplan–Meier analysis mirrored these findings, showing the highest cumulative incidence in the antiplatelet group and the lowest in the anticoagulant group without a significant difference overall (*p* = 0.061) (Figure 4B).

### 3.6 CRMB events

The rate of CRMB events was 2.5% (3/118) in the anticoagulant group, 1.4% (1/70) in the combination group, and 0% in the antiplatelet group (anticoagulant vs. antiplatelet: *p* = 1.000; combination vs. antiplatelet: *p* = 1.000; combination vs. anticoagulant: *p* = 1.000) (Figure 3D). Kaplan–Meier analysis indicated that the cumulative incidence of CRMB events was highest in the anticoagulant group, followed by the combination group, with the lowest incidence in the antiplatelet group (*p* = 0.358) (Figure 4C).

Given the low number of thromboembolic and CRMB events across groups, the Kaplan–Meier curves for these outcomes should be interpreted with caution.

### 3.7 Subgroup analysis

#### 3.7.1 Age (<65 years, ≥65 years)

**Figure 5:**
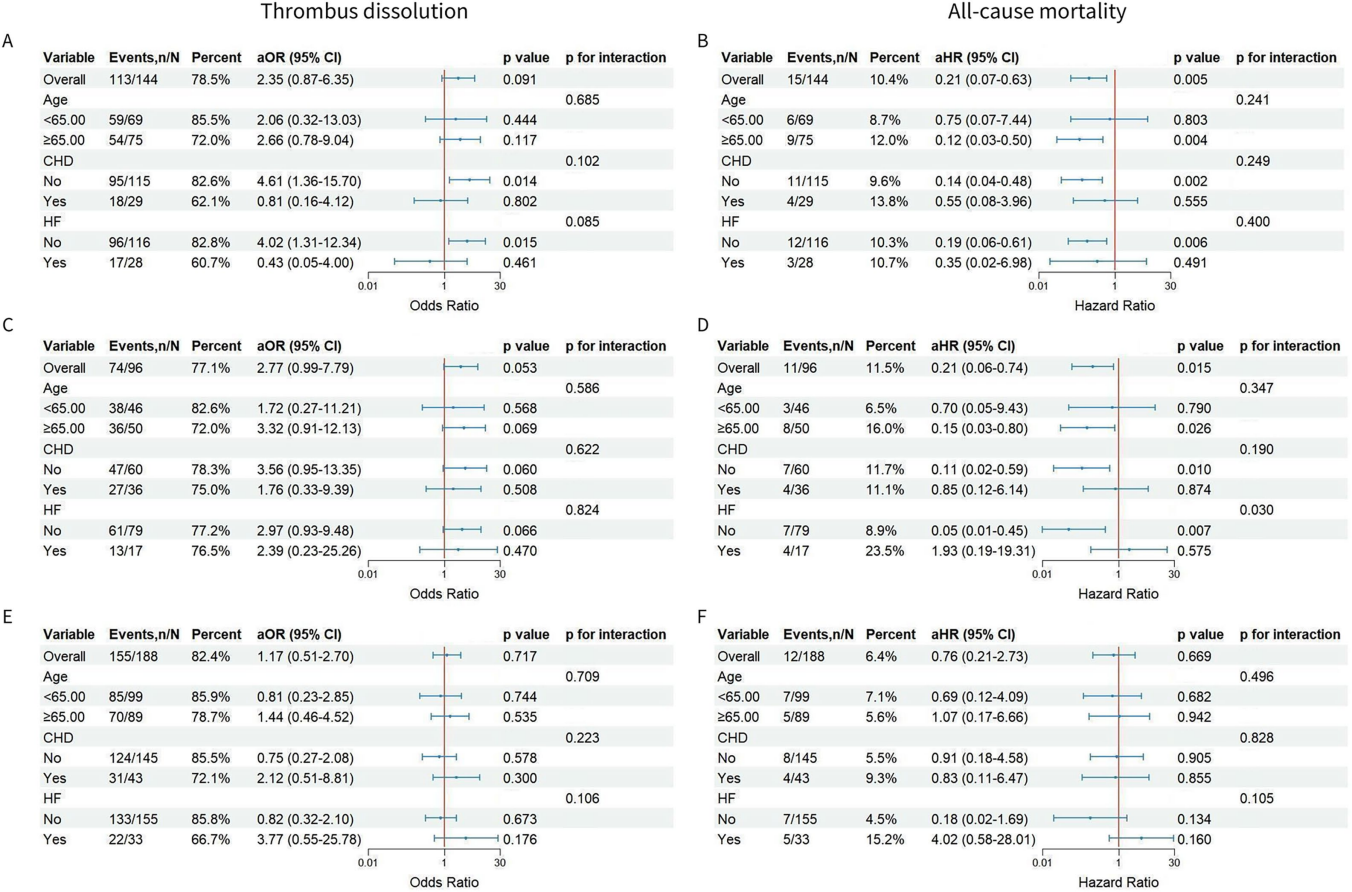
Forest plots of subgroup analysis adjusting for covariates. **(A)** (Anticoagulant vs. antiplatelet), **(C)** (combination vs. antiplatelet), and **(E)** (combination vs. anticoagulant) illustrated subgroup analyses for thrombus dissolution. **(B)** (Anticoagulant vs. antiplatelet), **(D)** (combination vs. antiplatelet), and **(F)** (combination vs. anticoagulant) illustrated subgroup analyses for all-cause mortality. The subgroup analysis was adjusted for age, coronary heart disease, and heart failure. CHD, coronary heart disease; HF, heart failure; aHR: adjusted hazard ratio; aOR: adjusted odds ratio; 95% CI: 95% confidence interval.

No significant interactions were observed between treatment groups and age with respect to thrombus dissolution or all-cause mortality, with all p-interactions >0.1 (Figure 5). In patients aged ≥65 years, both anticoagulant group and combination group were associated with a significantly lower risk of all-cause mortality compared with the antiplatelet group (anticoagulant: aHR 0.12, 95% CI 0.03–0.50, *p* = 0.004; combination: aHR 0.15, 95% CI 0.03–0.80, *p* = 0.026; Figures 5B,D).

#### 3.7.2 A history of CHD

No significant interactions were observed between treatment groups and a history of CHD regarding thrombus dissolution or all-cause mortality (all p-interactions >0.1; Figure 5). In patients without a history of CHD, the anticoagulant group significantly improved thrombus dissolution (aOR 4.61, 95% CI 1.36–15.70, *p* = 0.014; Figure 5A) and reduced the risk of all-cause mortality (aHR 0.14, 95% CI 0.04–0.48, *p* = 0.002; Figure 5B) compared to the antiplatelet group. Furthermore, the combination group exhibited a significant trend towards reduced all-cause mortality risk relative to the antiplatelet group (aHR 0.11, 95% CI 0.02–0.59, *p* = 0.010; Figure 5D).

#### 3.7.3 A history of HF

No significant interactions were observed between treatment groups and a history of HF regarding thrombus dissolution or all-cause mortality, except for a borderline interaction in the comparison of anticoagulant and antiplatelet therapy on thrombus dissolution (p-interaction = 0.085; Figure 5A) and a significant interaction for all-cause mortality between combination versus antiplatelet therapy (p-interaction = 0.030; Figure 5D). In patients without HF, combination therapy was associated with a marked reduction in all-cause mortality compared with antiplatelet therapy (aHR 0.05, 95% CI 0.01–0.45, *p* = 0.007; Figure 5D). Similarly, the anticoagulant group was associated with both an increased likelihood of thrombus dissolution (aOR 4.02, 95% CI 1.31–12.34, *p* = 0.015; Figure 5A) and a reduced risk of all-cause mortality (aHR 0.19, 95% CI 0.06–0.61, *p* = 0.006; Figure 5B) compared with the antiplatelet group.

## 4 Discussion

To our knowledge, real-world evidence directly comparing the efficacy and safety of different antithrombotic regimens in patients with intracardiac thrombus remains limited, and the present study provides additional data in this clinical context. Our findings reveal that anticoagulant monotherapy was associated with a lower one-year all-cause mortality compared with antiplatelet monotherapy, whereas its effect on thrombus resolution appeared heterogeneous across different thrombus locations. Importantly, combination therapy demonstrated no additional benefits over either antiplatelet or anticoagulant monotherapy, suggesting that the routine addition of antiplatelet agents to anticoagulation may not provide incremental benefit. Taken together, these findings suggest that anticoagulant-based strategies may represent a reasonable therapeutic option for intracardiac thrombus management in routine clinical practice. However, given the observational design and relatively small sample size, prospective randomized trials are needed to confirm the optimal antithrombotic strategy, clarify patient selection criteria, and establish long-term outcomes beyond 1 year.

Intracardiac thrombus is commonly encountered in clinical practice, particularly among patients with cardiovascular diseases, who are at an elevated risk of all-cause mortality and adverse cardiovascular events. For instance, Lattuca et al. reported that 37.1% of 159 patients with LVT experienced severe adverse cardiovascular events, and 18.9% suffered mortality (8). Current treatment strategies for intracardiac thrombus primarily encompass antithrombotic therapy and thrombus removal procedures. However, invasive thrombus removal may be associated with high costs, residual thrombus burden, and increased risks of cardiovascular death and hospital readmissions (17). Therefore, antithrombotic therapy remains the cornerstone of treatment, highlighting the necessity for optimal strategies to improve clinical outcomes and reduce mortality. Guideline recommendations are more established for LVT than for other intracardiac thrombus locations. Existing LVT guidance recommends OACs for persistent LVT, and emerging evidence indicates that direct oral anticoagulants (DOACs) may be reasonable alternatives to warfarin (6). Additionally, STEMI guidance recommends considering OACs for up to 6 months after an LVT diagnosis (7). Nevertheless, real-world practice demonstrates substantial variability in antithrombotic management. Prior studies indicated that a considerable proportion of patients with LVT receive antiplatelet therapy alone or in combination with anticoagulation. For instance, a retrospective analysis of 909 patients with LVT reported that 40.6% received antiplatelet medication alone (18), while another study involving 92 patients found that 79 patients received combination therapy with anticoagulants and antiplatelet drugs (10). For patients with AF and LAA thrombus, the 2024 ESC guidelines for AF recommend initiating therapeutic anticoagulation and continuing treatment for at least 4 weeks, with follow-up imaging (e.g., TOE) to confirm thrombus resolution (19). Beyond this specific clinical scenario, current guidelines provide limited guidance on the comparative antithrombotic management of intracardiac thrombus across different locations. Available retrospective evidence suggests that oral anticoagulants are commonly used in patients with LA or LAA thrombus (20). Importantly, the 2024 ESC guidelines also discourage routine combination of OACs with antiplatelet therapy solely for thromboembolic risk reduction in AF, and restrict such combination therapy to selected patients with acute vascular indications (19). However, in routine practice, clinicians may still employ combination therapy in selected patients with competing ischemic indications (e.g., concomitant CHD, recent stenting, or coexisting LVT), which likely contributes to the observed heterogeneity in antithrombotic strategies. Collectively, these considerations highlight ongoing uncertainty and practice variation, reinforcing the need to better clarify the role of different antithrombotic strategies across intracardiac thrombus locations.

In this study, compared with the antiplatelet therapy, anticoagulant therapy was associated with higher rates of thrombus resolution in patients with intracardiac thrombus; however, this effect appeared to vary across different thrombus locations. Notably, thrombus resolution in the anticoagulation group appeared to be numerically higher in patients with left atrial appendage (LAA) thrombus than in those with atrial or left ventricular thrombus. This heterogeneity may be related to differences in the underlying pathophysiological mechanisms of thrombus formation across cardiac chambers rather than definitive treatment effects. Approximately 90% of LAA thrombi are associated with atrial fibrillation (AF), a condition in which blood stasis and activation of the coagulation cascade play central roles (21, 22). Anticoagulant therapy may have greater etiological relevance for LAA thrombi related to AF-associated hypercoagulability and blood flow stasis (23). By contrast, no clear additional benefit was observed with combination therapy compared with anticoagulant or antiplatelet monotherapy, which may reflect more conservative anticoagulant dosing in the combination group due to concerns regarding bleeding risk. Our findings are generally consistent with prior studies supporting the role of anticoagulant therapy in thrombus resolution, although reported resolution rates vary across populations and study designs. A prospective, single-arm study of 64 patients with LVT reported a 95.3% rate of thrombus dissolution or reduction after 12 weeks of rivaroxaban therapy (24). Similarly, a retrospective single-center study showed a 53% resolution rate of LA or LAA thrombus following anticoagulant treatment (20), while another study of 90 patients with atrial thrombus treated with OACs reported a complete resolution rate of 60% after a median follow-up of 4 months (13). Heart failure (HF) has been recognized as an important factor associated with persistent intracardiac thrombus, potentially due to blood stasis resulting from impaired myocardial contractility and endothelial injury related to myocardial inflammation or necrosis (13, 25). In our subgroup analyses, the apparent therapeutic benefit of anticoagulant therapy was more pronounced in patients without a history of HF, where the absence of blood stasis and myocardial injury may facilitate thrombus resolution (25). Likewise, anticoagulant therapy was associated with higher thrombus resolution rates in patients without CHD. These observations suggest that differences in dominant pathophysiological mechanisms of thrombus formation across patient subgroups may influence treatment response. In patients with CHD, platelet activation plays a central role in atherosclerotic arterial thrombosis (26), whereas intracardiac thrombus formation in patients without CHD or HF is more likely driven by coagulation cascade activation and blood flow stasis. Beyond their role in primary hemostasis, platelets actively participate in inflammatory and immunomodulatory processes that contribute to atherosclerotic plaque progression and destabilization (27). Accordingly, antiplatelet agents are particularly effective in clinical settings dominated by platelet-driven atherothrombosis. However, intracardiac thrombus differs fundamentally from atherothrombotic disease, and antiplatelet therapy alone may be insufficient to prevent thrombus propagation in this setting. In contrast, anticoagulant agents directly target key components of the coagulation pathway and are therefore better suited to counteract thrombus formation driven by blood stasis and hypercoagulable states (28). Nevertheless, antithrombotic decision-making in real-world clinical practice is often more complicated. Abnormalities in platelet count, especially thrombocytopenia, are frequently encountered in patients with thrombotic disorders and pose a significant clinical challenge due to the concurrent risks of bleeding and thrombosis. The emergence of platelet-modulating therapies, such as thrombopoietin receptor agonists (29), further illustrates the complexity of managing patients with concomitant thrombocytopenia and thrombotic disease. Although these agents were not evaluated in the present study and are not indicated for intracardiac thrombus, their increasing use highlights the need for future prospective studies with well-defined patient selection criteria to optimize antithrombotic strategies in heterogeneous populations.

In addition to enhancing thrombus dissolution, anticoagulant therapy also significantly reduced all-cause mortality. Our findings align with earlier research, which demonstrated that patients receiving anticoagulant therapy had a lower all-cause mortality rate compared to those who did not, although the difference was not statistically significant (18). Another study involving 97 patients with right-sided intracardiac thrombus reported higher survival rates associated with anticoagulant therapy (30). In our study, there was no significant difference in mortality risk between the combination and anticoagulant therapies. However, subgroup analyses further showed that anticoagulant-based regimens were linked to a lower all-cause mortality rate compared to antiplatelet therapy, particularly in patients aged ≥65 years. This may reflect greater benefit from anticoagulation in older individuals, who are more vulnerable to fatal bleeding complications from antiplatelet use (31, 32). In our subgroup analysis of LVT, we found that compared with antiplatelet therapy, the combination treatment was a favorable factor in reducing all-cause mortality. This might be because oral anticoagulation therapy is the cornerstone of LVT treatment. Anticoagulant therapy is associated with a higher rate of LVT dissolution and a lower incidence of embolic events, thus being related to a lower mortality rate (33). Patients without CHD benefited significantly from anticoagulant-based regimens, indicating potential superiority over antiplatelet therapy in this subgroup. Patients without CHD experienced greater mortality reduction, possibly due to differing underlying thrombotic mechanisms. Patients with CHD are more prone to arterial thrombosis resulting from atherosclerotic plaque rupture and typically rely on antiplatelet therapy (26). Conversely, anticoagulants are especially beneficial in patients without CHD, who often have thrombus formation driven by coagulation system abnormalities or venous thrombotic processes (34). Moreover, in patients without HF, both anticoagulant and combination therapies markedly reduced mortality. These patients, typically with better cardiac function and fewer comorbidities, responded more positively to anticoagulant therapy, which effectively prevented thrombus formation and reduced cardiac-related complications. The presence of multiple organ dysfunctions in HF patients, however, may mitigate the benefits of anticoagulant therapy (35, 36). These findings underscore the importance of individualized therapy based on specific patient characteristics and underlying mechanisms of thrombus formation.

Antiplatelet and anticoagulant therapies prevent thrombus formation via distinct mechanisms: antiplatelets block platelet activation pathways and anticoagulants inhibit clotting factors (37). However, their efficacy in reducing thromboembolic events yielded mixed results across studies. Zhou et al. identified discontinuation of anticoagulation as an independent risk factor for LVT recurrence, with continued anticoagulant therapy reducing the risk of systemic embolism (38). In contrast, Lee et al. found no significant difference in post-treatment thromboembolism rates between patients receiving anticoagulant or antiplatelet therapies for LVT (9). And Shi et al. reported similar rates of systemic embolism in the anticoagulation (5.3%) and non-anticoagulation groups (4.8%) (18). Our study demonstrated that thromboembolic event rates were higher in the antiplatelet group (11.5%) compared to the anticoagulant group (3.4%) and the combination group (4.3%), with no statistically significant differences.

While anticoagulant and combination therapies are crucial in managing intracardiac thrombus, the risk of CRMB remains a significant concern. In a retrospective analysis, 4.8% of patients with LVT on anticoagulant therapy experienced CRMB, compared to none in those treated with antiplatelet therapy, though this difference was not statistically significant (9). Similarly, a single-center retrospective study reported CRMB rates of 1.2% in the anticoagulant group and 2.3% in the non-anticoagulant group (92.5% of whom were on antiplatelet therapy), again showing no significant differences between the groups (18). Our study aligns with these results, reporting CRMB incidences of 2.5% in the anticoagulant group, 1.4% in the combination group, and no CRMB events in the antiplatelet group. Importantly, the differences in CRMB rates across the three groups were not statistically significant.

Our study has several limitations. First, given its retrospective, single-center nature and relatively small sample size, the statistical power was limited, increasing the risk of type II error. Treatment allocation was determined by real-world clinical decision-making rather than random assignment, raising the possibility of confounding. To mitigate this issue, patients who underwent thrombectomy during the index hospitalization were excluded from the analysis, as procedural intervention would substantially alter post-procedural antithrombotic regimens and could independently influence clinical outcomes. Although we adjusted for multiple clinically relevant covariates, several important factors were not systematically available in this retrospective dataset. These include detailed thrombus characteristics (such as size, morphology, and mobility), and patient adherence to antithrombotic therapy during follow-up. Second, heterogeneity in thrombus location may have influenced treatment effects. Although thrombus type was incorporated into multivariable models, residual confounding related to thrombus-location heterogeneity cannot be entirely excluded, particularly given the limited number of outcome events within individual thrombus-type strata. Third, there was substantial variability in antithrombotic regimens, especially regarding the type and dosing of anticoagulants. The increasing off-label use of DOACs for intracardiac thrombus further underscores the necessity for large-scale prospective studies or randomized controlled trials to compare vitamin K antagonists (VKAs) with DOACs and to define optimal antithrombotic strategies in this population. Fourth, the primary endpoint of this study is all-cause mortality. Due to the limitations of the data source, we cannot reliably classify the causes of death (such as cardiovascular deaths and non-cardiovascular deaths). Therefore, the results of this study mainly reflect the impact of the intervention measures on the overall survival of patients, but cannot make a clear inference about the specific mechanism of reducing the risk of cardiovascular deaths.

## 5 Conclusion

In summary, our study suggests that anticoagulant monotherapy was associated with a lower one-year all-cause mortality compared with antiplatelet therapy in patients with intracardiac thrombus. With respect to thrombus dissolution, the effect of anticoagulant therapy appeared to differ across thrombus locations, with numerically higher resolution rates observed in patients with LAA thrombus than in those with atrial or left ventricular thrombus. Given that no clear additional benefits were observed with combination therapy compared with anticoagulant or antiplatelet monotherapy, these findings indicate that anticoagulant monotherapy may represent a reasonable therapeutic option for the management of intracardiac thrombus in clinical practice.

## Data Availability

The original contributions presented in the study are included in the article/Supplementary material, further inquiries can be directed to the corresponding authors.
