# Supplementary material for: Comparative efficacy and safety of anticoagulant, combination, and antiplatelet therapies in patients with intracardiac thrombus: a real-world retrospective study
Source: Front Med (Lausanne). 2026 Feb 18;13:1723890. doi: 10.3389/fmed.2026.1723890 (PMC12956654; doi:10.3389/fmed.2026.1723890)
Supplement: Supplementary file 1 [file Data_Sheet_1.docx]

Supplemental Materials

**Supplemental Table 1** Medication regimens in the antiplatelet group (N = 26)

| Medication regimens | | n (%) |
| --- | --- | --- |
| SAPT | aspirin 100 mg qd | 9 (34.6%) |
|  | clopidogrel 50 mg qd | 2 (7.7%) |
|  | clopidogrel 75 mg qd | 7 (26.9%) |
| DAPT | aspirin 100 mg qd + clopidogrel 75 mg qd | 3 (11.5%) |
|  | aspirin 100 mg qd + ticagrelor 90 mg bid | 5 (19.2%) |

Abbreviations: SAPT, single antiplatelet therapy; DAPT, dual antiplatelet therapy; qd, once daily; bid, twice daily.

**Supplemental Table 2** Medication regimens in the anticoagulant group (N = 118)

| Medication regimens | | n (%) |
| --- | --- | --- |
| warfarin | 1.25mg qd | 1 (0.8%) |
|  | 1.5mg qd | 2 (1.7%) |
|  | 2.25mg qd | 5 (4.2%) |
|  | 2.5mg qd | 2 (1.7%) |
|  | 3mg qd | 17 (14.4%) |
|  | 3.125mg qd | 1 (0.8%) |
|  | 3.75mg qd | 5 (4.2%) |
|  | 4.5mg qd | 5 (4.2%) |
|  | 6mg qd | 1 (0.8%) |
| rivaroxaban | 10 mg qd | 1 (0.8%) |
|  | 15 mg qd | 18 (15.3%) |
|  | 20 mg qd | 42 (35.6%) |
|  | 10 mg bid | 1 (0.8%) |
| dabigatran | 110 mg bid | 7 (5.9%) |
|  | 150 mg bid | 7 (5.9 %) |
| edoxaban | 15 mg qd | 1 (0.8%) |
|  | 30 mg qd | 2 (1.7%) |

Abbreviations: qd, once daily; bid, twice daily.

**Supplemental Table 3** Medication regimens in the combination group (N = 70)

| Medication regimens | | All | SAPT | DAPT |
| --- | --- | --- | --- | --- |
|  |  | N = 70 | N = 40 | N = 30 |
| warfarin | 1.5mg qd | 2 (2.9%) | 2 (5.0%) | - |
|  | 2.5mg qd | 1 (1.4%) | 1 (2.5%) | - |
|  | 3mg qd | 4 (5.7%) | 3 (7.5%) | 1 (3.3%) |
|  | 4.5mg qd | 4 (5.7%) | 2 (5.0%) | 2 (6.7%) |
| rivaroxaban | 5 mg qd | 1 (1.4%) | 1 (2.5%) | - |
|  | 10 mg qd | 4 (5.7%) | 2 (5.0%) | 2 (6.7%) |
|  | 15 mg qd | 34 (48.6%) | 17 (42.5%) | 17 (56.7%) |
|  | 20 mg qd | 2 (2.9%) | 2 (5.0%) | - |
|  | 10 mg bid | 2 (2.9%) | 2 (5.0%) | - |
|  | 15 mg bid | 1 (1.4%) | 1 (2.5%) | - |
| dabigatran | 110 mg bid | 9 (12.9%) | 2 (5.0%) | 7 (23.3%) |
|  | 150 mg bid | 2 (2.9%) | 1 (2.5%) | 1 (3.3%) |
| edoxaban | 30 mg qd | 2 (2.9%) | 2 (5.0%) | - |
|  | 60mg qd | 2 (2.9%) | 2 (5.0%) | - |

Abbreviations: SAPT, single antiplatelet therapy; DAPT, dual antiplatelet therapy; qd, once daily; bid, twice daily.

**Supplemental Table 4** The thrombus dissolution rates of atrial thrombus, LV thrombus and LAA thrombus among the treatment groups

| Group | Thrombus types | Thrombus dissolution rates | p value | |
| --- | --- | --- | --- | --- |
| Antiplatelet group | Atrial thrombus | 0.0% (0/0) | Ref | - |
|  | LV thrombus | 62.5% (15/24) | 1.000 | Ref |
|  | LAA thrombus | 50.0% (1/2) | 1.000 | 1.000 |
| Anticoagulant group | Atrial thrombus | 67.6% (23/34) | Ref | - |
|  | LV thrombus | 79.5% (31/39) | 0.377 | Ref |
|  | LAA thrombus | 95.6% (43/45) | < 0.001 | 0.039 |
| Combination group | Atrial thrombus | 100.0% (5/5) | Ref | - |
|  | LV thrombus | 81.0% (51/63) | 0.577 | Ref |
|  | LAA thrombus | 100.0% (2/2) | 1.000 | 1.000 |

**Supplemental Table 5** The thrombus dissolution rates in each group of atrial thrombus, LV thrombus and LAA thrombus.

| Thrombus types | Group | Thrombus dissolution rates | p value | |
| --- | --- | --- | --- | --- |
| Atrial thrombus | Antiplatelet group | 0.0% (0/0) | Ref | - |
|  | Anticoagulant group | 67.6% (23/34) | 1.000 | Ref |
|  | Combination group | 100% (5/5) | 1.000 | 0.296 |
| LV thrombus | Antiplatelet group | 62.5% (15/24) | Ref | - |
|  | Anticoagulant group | 79.5% (31/39) | 0.237 | Ref |
|  | Combination group | 81.0% (51/63) | 0.129 | 1.000 |
| LAA thrombus | Antiplatelet group | 50.0% (1/2) | Ref | - |
|  | Anticoagulant group | 95.6% (43/45) | 0.125 | Ref |
|  | Combination group | 100% (2/2) | 1.000 | 1.000 |

**Supplemental Table 6** The All-cause mortality rates of atrial thrombus, LV thrombus and LAA thrombus among the treatment groups

| Group | Thrombus types | All-cause mortality rates | p value | |
| --- | --- | --- | --- | --- |
| Antiplatelet group | Atrial thrombus | 0.0% (0/0) | Ref | - |
|  | LV thrombus | 25.0% (6/24) | 1.000 | Ref |
|  | LAA thrombus | 50.0% (1/2) | 1.000 | 0.474 |
| Anticoagulant group | Atrial thrombus | 11.8% (4/34) | Ref | - |
|  | LV thrombus | 7.7% (3/39) | 0.698 | Ref |
|  | LAA thrombus | 2.2% (1/45) | 0.159 | 0.333 |
| Combination group | Atrial thrombus | 0.0% (0/5) | Ref | - |
|  | LV thrombus | 6.3% (4/63) | 1.000 | Ref |
|  | LAA thrombus | 0.0% (0/2) | 1.000 | 1.000 |

**Supplemental Table 7** The All-cause mortality rates in each group of atrial thrombus, LV thrombus and LAA thrombus.

| Thrombus types | Group | All-cause mortality rates | p value | |
| --- | --- | --- | --- | --- |
| Atrial thrombus | Antiplatelet group | 0.0% (0/0) | Ref | - |
|  | Anticoagulant group | 11.8% (4/34) | 1.000 | Ref |
|  | Combination group | 0.0% (0/5) | 1.000 | 1.000 |
| LV thrombus | Antiplatelet group | 25.0% (6/24) | Ref | - |
|  | Anticoagulant group | 7.7% (3/39) | 0.073 | Ref |
|  | Combination group | 6.3% (4/63) | 0.024 | 1.000 |
| LAA thrombus | Antiplatelet group | 50.0% (1/2) | Ref | - |
|  | Anticoagulant group | 2.2% (1/45) | 0.084 | Ref |
|  | Combination group | 0.0% (0/2) | 1.000 | 1.000 |

**Supplemental Table 8** Univariate regression analysis of thrombus dissolution and all-cause mortality in each group of LV thrombus.

| Group | Thrombus dissolution | | All-cause mortality | |
| --- | --- | --- | --- | --- |
|  | Univariate analysis | | Univariate analysis | |
|  | OR (95% CI) | p value | HR (95% CI) | p value |
| Part A：Antiplatelet group as the reference | | | | |
| Antiplatelet group | Ref | - | Ref | - |
| Anticoagulant group | 2.33 (0.75 - 7.23) | 0.145 | 0.28 (0.07 - 1.12) | 0.072 |
| Combination group | 2.55 (0.90 - 7.20) | 0.077 | 0.23 (0.06 - 0.80) | 0.021 |
| Part B：Anticoagulant group as the reference | | | | |
| Anticoagulant group | Ref | - | Ref | - |
| Combination group | 1.10 (0.40 - 2.98) | 0.856 | 0.80 (0.18 - 3.55) | 0.765 |

Abbreviations: OR: odds ratio; HR: hazard ratio; 95% CI: 95% confidence interval.


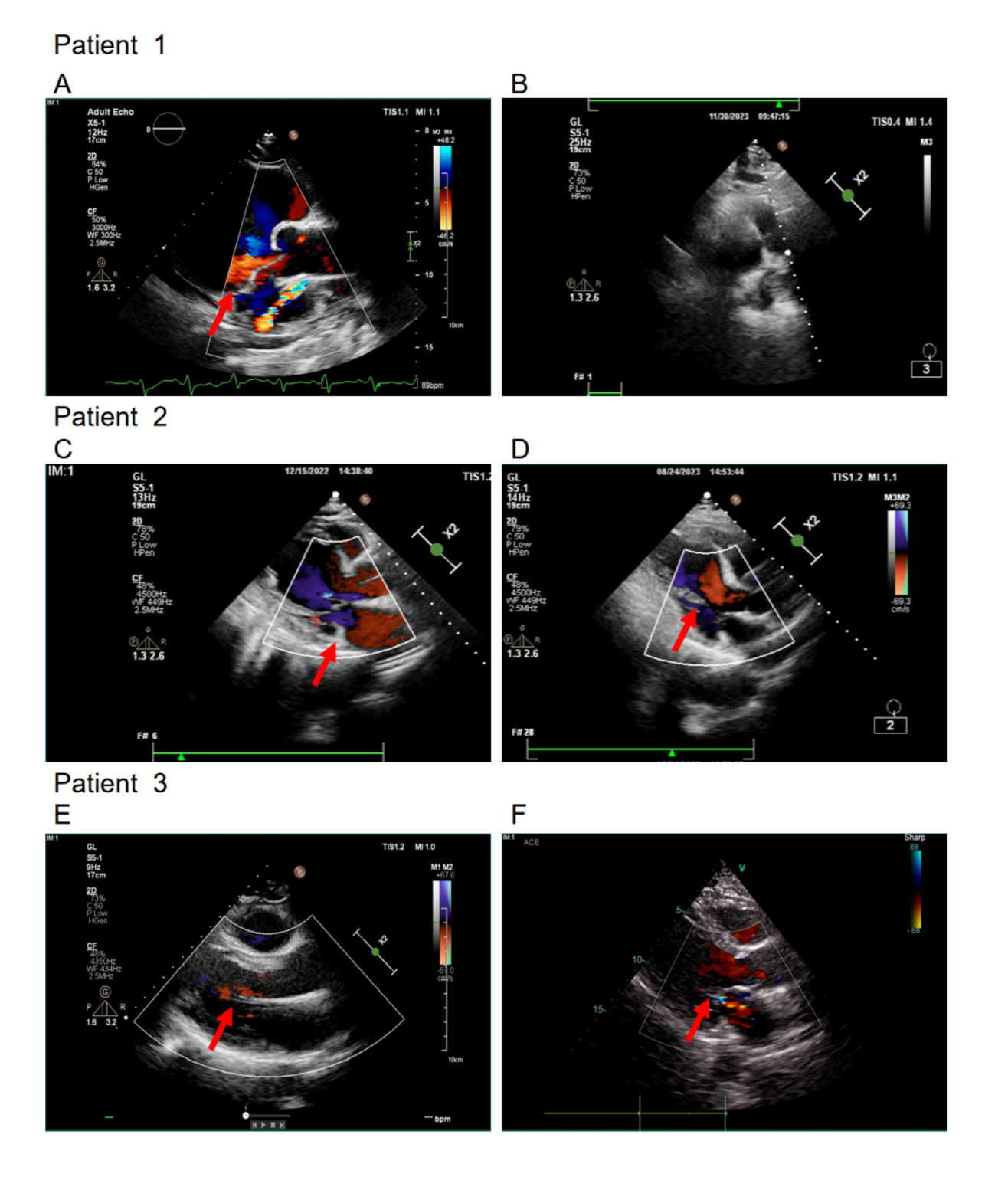


**Supplemental Fig.1** Patient examples of echocardiographic images for intracardiac thrombus at baseline (A, C, E) and follow-up visits (B, D, F). Patient 1 had thrombus dissolution (B) compared to intracardiac thrombus (red arrows) at baseline (A). Patient 2 had thrombus reduced (D) compared to intracardiac thrombus (red arrows) at baseline (C). Patient 3 had thrombus increased (F) compared to intracardiac thrombus (red arrows) at baseline (E). Follow-up period: one year.
